# Plasma Biomarkers to Detect Prevalent or Predict Progressive Tuberculosis Associated With Human Immunodeficiency Virus–1

**DOI:** 10.1093/cid/ciy823

**Published:** 2018-09-26

**Authors:** Maia Lesosky, Molebogeng X Rangaka, Cara Pienaar, Anna K Coussens, Rene Goliath, Shaheed Mathee, Judith Mwansa-Kambafwile, Gary Maartens, Robert J Wilkinson, Katalin Andrea Wilkinson

**Affiliations:** 1 Division of Epidemiology & Biostatistics, School of Public Health and Family Medicine; 2 Wellcome Centre for Infectious Diseases Research in Africa, Institute of Infectious Diseases and Molecular Medicine, Observatory, South Africa; 3 Department of Medicine, Faculty of Health Sciences, University of Cape Town, Observatory, South Africa; 4 Institute for Global Health, Faculty of Population Health Sciences, University College London, United Kingdom; 5 Department of Pathology, Faculty of Health Sciences, University of Cape Town, Observatory; 6 Site B Khayelitsha Community Health Centre, Western Cape Department of Health, South Africa; 7 Department of Medicine, Imperial College London, London, United Kingdom; 8 The Francis Crick Institute, London, United Kingdom

**Keywords:** tuberculosis, HIV-1, predictive, plasma, biomarker

## Abstract

**Background:**

The risk of individuals infected with human immunodeficiency virus (HIV)-1 developing tuberculosis (TB) is high, while both prognostic and diagnostic tools remain insensitive. The potential for plasma biomarkers to predict which HIV-1–infected individuals are likely to progress to active disease is unknown.

**Methods:**

Thirteen analytes were measured from QuantiFERON Gold in-tube (QFT) plasma samples in 421 HIV-1–infected persons recruited within the screening and enrollment phases of a randomized, controlled trial of isoniazid preventive therapy. Blood for QFT was obtained pre-randomization. Individuals were classified into prevalent TB, incident TB, and control groups. Comparisons between groups, supervised learning methods, and weighted correlation network analyses were applied utilizing the unstimulated and background-corrected plasma analyte concentrations.

**Results:**

Unstimulated samples showed higher analyte concentrations in the prevalent and incident TB groups compared to the control group. The largest differences were seen for C-X-C motif chemokine 10 (CXCL10), interleukin-2 (IL-2), IL-1α, transforming growth factor-α (TGF-α). A predictive model analysis using unstimulated analytes discriminated best between the control and prevalent TB groups (area under the curve [AUC] = 0.9), reasonably well between the incident and prevalent TB groups (AUC > 0.8), and poorly between the control and incident TB groups. Unstimulated IL-2 and IFN-γ were ranked at or near the top for all comparisons, except the comparison between the control vs incident TB groups. Models using background-adjusted values performed poorly.

**Conclusions:**

Single plasma biomarkers are unlikely to distinguish between disease states in HIV-1 co-infected individuals, and combinations of biomarkers are required. The ability to detect prevalent TB is potentially important, as no blood test hitherto has been suggested as having the utility to detect prevalent TB amongst HIV-1 co-infected persons.

## Teaser

In prevalent tuberculosis (TB), incident TB, and control samples, we evaluated 13 plasma biomarkers to identify individuals infected with human immunodeficiency virus–1 who were susceptible to active TB: biomarkers differentiated prevalent TB from control samples, but not incident TB.

Tuberculosis (TB) associated with human immunodeficiency virus (HIV)-1 is a health burden in Africa, despite implementation of the World Health Organization’s directly-observed, short-course therapy strategy for TB; the widespread roll-out of combination antiretroviral therapy (ART); and guidelines to increase provision of isoniazid preventive therapy (IPT). The risk of developing TB in HIV-1–infected people exceeds that in HIV-1–uninfected people, even after accounting for risk reduction by ART [1–3]. HIV-1 co-infection impacts TB-specific immune responses, causing false negative results in immune tests of TB sensitization. Currently, testing for latent TB infection (LTBI) is performed by either Tuberculin skin testing (TST) or interferon gamma release assays (IGRA). HIV-1 infection impairs the sensitivity of both of these tests [4–6], underestimating those who would benefit from IPT, particularly amongst those newly commencing ART, leading to vulnerable patients potentially being untreated.

The role of IPT to decrease the risk of TB in HIV-1–infected people is well-recognized [7]. A randomized, controlled trial (RCT) of IPT plus ART versus ART alone for the prevention of TB in a large, well-characterized group of HIV-1–infected persons conducted in Khayelitsha, South Africa (ART-IPT Study), established that TST- or IGRA-negative, HIV-infected persons on ART also benefit from IPT [8]. Thus, current tests for latent TB imperfectly identify those likely to benefit from IPT. It is not known whether tests evaluating an extended spectrum of biomarkers in plasma would enhance the risk stratification of infected individuals, as studies assessing biomarkers other than interferon (IFN)-γ to predict active disease or potential benefits from IPT are lacking. There is an urgent need for tests that can distinguish prevalent TB from LTBI, as the fear of treating TB with IPT monotherapy is a major barrier to IPT implementation.

Here, we investigated the predictive performance of 13 preselected analytes determined from QuantiFERON Gold in-tube (QFT) plasma samples from HIV-1–infected persons screened for the ART-IPT Study. Patients were grouped into those with prevalent (active) TB that were screened out prior to RCT randomization; those who developed TB either after randomization or during the longitudinal follow-up (incident TB); and those who did not develop TB (controls). Analytes (Supplementary Table S1) were selected for their relevance in active TB and LTBI based on the available literature at the time of the study design, with the hypothesis that those biomarkers that are elevated at baseline in patients who either go on to have active TB (and thus should not start IPT) or go on to develop TB during longitudinal follow-up could be potential biomarkers predictive of TB.

## METHODS

### Study Design and Participants

This study was conducted within the screening and follow-up of a previously-reported, randomized, controlled trial [8]. Briefly, a pragmatic, individually-randomized, double-blind, placebo-controlled trial of IPT in adults with HIV-1 infection was conducted between 31 January 2008 and 31 September 2011. Patients were prescribed, or commencing, ART at the Ubuntu clinic in Khayelitsha, South Africa. Ethical approval was obtained from the University of Cape Town Faculty of Health Sciences Human Research Ethics Committee both for the trial (REC/REF: 013/2007) and for the laboratory sub-study (REC/REF: 245/2009). Written consent or a thumb-print was required from all participants prior to screening. A total of 421 persons were included in this analysis, based on IGRA sample availability at screening, and included 51 individuals who developed clinically- or microbiologically-confirmed TB during the 4-year duration of the study (incident TB). Prevalent TB (defined as sputum-culture positive) was identified in 87 individuals at the time of randomization (when culture results became available), who were referred for treatment, rendering them ineligible for the RCT. There were 283 controls with IGRA samples available and no signs or symptoms of TB during the duration of the follow-up (median duration of follow-up in the main study was 2.5 years), who were randomly chosen just before and just after the incident and prevalent cases in the order of recruitment. All samples used in the current analyses were collected before randomization.

### Definition of Tuberculosis Diagnoses

Cases labelled as prior TB had a previous treatment history that included diagnosis and treatment for active TB in a healthcare facility. There is no routine LTBI diagnosis in South Africa using either TST or IGRA, thus all cases labelled as prior TB had previously-treated, active TB. Where incident or prevalent TB was not microbiologically confirmed, TB was diagnosed clinically with a combination of symptoms and a chest X-ray.

### Tuberculin Skin Testing and Interferon Gamma Release Assays

The TST (2 TU RT23 purified-protein derivative, Statens Serum Institut, Copenhagen, Denmark) was administered on the volar aspect of the left forearm by personnel trained in its administration. The TST induration was recorded after 48–72 h by the ballpoint pen and ruler method. Phlebotomy for IGRA (QuantiFERON Gold-in-tube, Qiagen) was performed on the same day that the TST was administered and the blood draw preceded the placement of the purified-protein derivative. The IGRA were performed in a Qiagen-accredited laboratory and interpreted according to the manufacturer’s guidelines. Laboratory personnel were blinded to TST results and TB symptoms, signs, and culture results, while clinicians were blinded to IGRA results.

### Luminex Multiplex Assay for Cytokines and Chemokines

QFT plasma samples from unstimulated (Nil) and TB-specific, antigen-stimulated (Ag) tubes were stored at -80^o^C until batched analyses for the analytes listed in Supplementary Table 1. Undiluted (Ag and Nil) QFT plasma samples were assayed on 11 plates using customized MilliplexTM kits (Millipore, St Charles, MO) on the Bio-Plex platform (Bio-Rad Laboratories, Hercules, CA). The upper limit of detection for all analytes was 10 000 pg/ml, while the mean lower limit of detection from the 11 plates was as follows (all in pg/ml): epidermal growth factor (EGF), 8; IFN-α2, 6; interleukin (IL)-1α, 3; C-X-C motif chemokine 10 (CXCL10), 10; chemokine (C-C motif) ligand (CCL) 3, 7; CCL4, 3; soluble CD40 ligand (sCD40L), 16; transforming growth factor-α (TGF-α), 3; tumor necrosis factor (TNF)-α, 3; vascular endothelial growth factor (VEGF), 205; IFN-γ, 3; IL-2, 3; and IL-10, 3.

### Statistical Analysis

Raw out-of-range values were adjusted, by replacing all out-of-range values lower than the lowest detectable value with 0 (1027 instances) and replacing all out-of-range values higher than the highest detectable value with 10 000 pg/mL (top standard, 13 instances) and adding the mean limit of detection per analyte to all plates. Results are presented for unstimulated (Nil) and stimulated-nil (Ag-Nil) values. Analyte values are nearly always presented in a log2-transformed scale as log2 pg/ml. Subgroups for analysis included the incident TB group, prevalent TB group, control group, and the combined incident and prevalent TB groups (TB-combined). Frequency (percent) or median (inter-quartile range) were calculated by group for discrete and continuous values, respectively. Sensitivity analyses were undertaken using the subgroup of culture-confirmed incident TB cases, the subgroup randomized to placebo, and the subgroup of prevalent TB cases who were smear-negative at baseline (smear-negative). The statistical tests used to compare groups were the Fisher’s exact test and Wilcoxon rank sum test, as appropriate. Throughout, a nominal threshold for statistical significance was set at α = .05, and false discovery rate correction (FDR) by Benjamini-Hochberg [9] was applied. These values are reported as *P* corrected. Data visualization was used to clarify differences between and within groups. A weighted correlation network analysis was carried out on the nil and background-corrected analyte levels, stratified by TB status, and presented with correlation diagrams. Correlations were estimated using Pearson’s correlation of the log2-transformed data.

Supervised learning models were applied to the data to predict class membership (eg, incident vs prevalent TB) in 2-way classifications. In all cases, analyte values were centered and scaled prior to input and all models were carried out with 10-fold cross-validation resampling to estimate classification accuracy. Sampling was further stratified by down-sampling to ensure balanced class representations in the re-samples. Training for the prediction models utilized a grid approach over model parameters with a specified grid length. A variable importance score, calculated as a scaled beta coefficient, was used as the primary means of determining an individual analyte’s impact on the classification outcome. The classification learners assessed included random forests to set a performance ceiling [10] and elastic-net regularization (glmnet) [11] for a potentially interpretable and applicable model. Models were selected on the basis of the largest minimum unbiased area under the curve (AUC) estimate. Lists of analytes and associated variable importance scores are presented. Receiver-operator curves were generated using the predicted vs observed classifications for each independent model and were graphed. Additional detailed methods are available in the online Supplementary Material.

## RESULTS

A total of 421 individuals were included in the analysis, with a median of 61 weeks (interquartile range, 28–91) to the onset of TB in the incident group. Characteristics did not vary between the parent screening population and the analysis sample (Supplementary Table S2). Between-group characteristics were similar for basic demographics and show expected differences for TB disease–related measures that were used as part of the screening or group definition in the RCT, with higher rates of symptoms, QFT, and/or TST positivity in the incident TB and prevalent TB groups than in the control group (Table 1). The proportion with previously-treated, active TB was higher in the control group compared to the TB-combined, incident TB, and prevalent TB groups (48% vs 35%, 41% and 32%, respectively; overall *P* = .028), and a higher proportion of the controls had previous exposure to ART. The control group also had a lower proportion of individuals with cluster of differentiation (CD) 4 <200 copies/mL compared to the TB-combined group (40% vs 61%, respectively; overall *P* < .001). In the incident TB group, 37% (19/51) were confirmed as culture positive during follow-up; those not culture-confirmed were diagnosed clinically (symptoms and a chest X-ray), due to the diagnosis occurring at a site other than the study site.

**Table 1.** Demographic Characteristics at Baseline and Culture Status as Confirmed During Study for Control, Incident TB, Prevalent TB, TB-combined, TB-Culture–positive, and Smear-negative Groups

Variable	Level	Control	Incident	Prevalent	Combined^a^	Culture-positive^b^	Smear-negative^c^
		(n = 283)	(n = 51)	(n = 87)	(n = 138)	(n = 106)	(n = 79)
**BMI, median (IQR)**		26 (23, 31)	24 (22, 30)	23 (20, 25)	24 (21, 27)	24 (20, 25)	24 (21, 25)
**Age, in years, median (IQR)**		34 (30, 39)	33 (30, 38)	35 (30, 39)	34 (30, 39)	34 (30, 38)	35 (30, 39)
**Follow-up time, in months,** ^**d**^ **median (IQR)**		13 (12, 14)	14 (6, 21)	…	…	…	…
**Sex**	**Male**	67 (24)	12 (24)	27 (31)	39 (28)	32 (30)	22 (28)
	**Female**	216 (76)	39 (76)	60 (69)	99 (72)	74 (70)	57 (72)
**Known prior TB**	**No**	146 (52)	29 (59)	59 (67)	88 (65)	66 (63)	55 (70)
	**Yes**	136 (48)	20 (41)	28 (32)	48 (35)	39 (37)	24 (30)
**TST positive at 5 mm**	**No**	160 (60)	25 (57)	30 (40)	55 (46)	36 (40)	26 (37)
	**Yes**	107 (40)	19 (43)	46 (61)	65 (54)	54 (60)	44 (63)
**TST positive at 10 mm**	**No**	168 (63)	25 (57)	32 (42)	57 (48)	38 (42)	28 (40)
	**Yes**	99 (37)	19 (43)	44 (58)	63 (52)	52 (58)	42 (60)
**TB symptoms at baseline**	**No**	265 (94)	43 (84)	52 (60)	95 (69)	68 (64)	48 (61)
	**Yes**	18 (6)	8 (16)	35 (40)	43 (31)	38 (36)	31 (39)
**CD4 category**	**< 200**	112 (40)	25 (49)	59 (69)	84 (61)	68 (65)	53 (68)
	**200–350**	85 (30)	15 (29)	14 (16)	29 (21)	20 (19)	13 (17)
	**350 +**	86 (30)	11 (22)	13 (15)	24 (18)	17 (16)	12 (15)
**Ever ART**	**No**	93 (36)	21 (44)	55 (65)	76 (57)	61 (59)	23 (79)
	**Yes**	164 (64)	27 (56)	30 (35)	57 (43)	42 (41)	6 (21)
**ART <3 months at baseline if ART**	**No**	145/164 (88)	27/27 (100)	24/30 (80)	51/57 (90)	36/42 (86)	48 (62)
	**Yes**	19/164 (12)	0 (0)	6/30 (20)	6/57 (10)	6/42 (14)	29 (38)
**QFT status at baseline** ^**e**^	**Negative**	155 (55)	27 (53)	30 (34)	57 (41)	38 (36)	26 (33)
	**Positive**	127 (45)	24 (47)	57 (66)	81 (59)	68 (64)	53 (67)
**Culture-confirmed TB**	**No**	283 (100)	32 (63)	0 (0)	32 (23)	0 (0)	0 (0)
	**Yes**	0 (0)	19 (37)	87 (100)	106 (77)	106 (100)	79 (100)
**INH** **treatment**	**No**	148 (53)	33 (65)	…	…	…	…
	**Yes**	135 (48)	18 (35)	…	…	…	…

Values are given as frequency (%) unless otherwise specified.

Abbreviations: ART, antiretroviral therapy; BMI, body mass index; INH, isoniazid; IQR, interquartile range; QFT, QuantiFERON Gold-in-tube; TB, tuberculosis; TST, Tuberculin skin testing.

^a^The combined column includes both the TB incident and TB prevalent groups.

^b^Prevalent and incident cases with positive culture confirmations.

^c^The smear-negative group is the subgroup of TB prevalent cases that were also smear-negative.

^d^Time from screening date until study drug stop date (controls) or date of TB diagnosis (incident TB).

^e^There was 1 individual from the control group who had an indeterminate QFT status at baseline.

### Analytes From Unstimulated Samples

**Figure 1.**
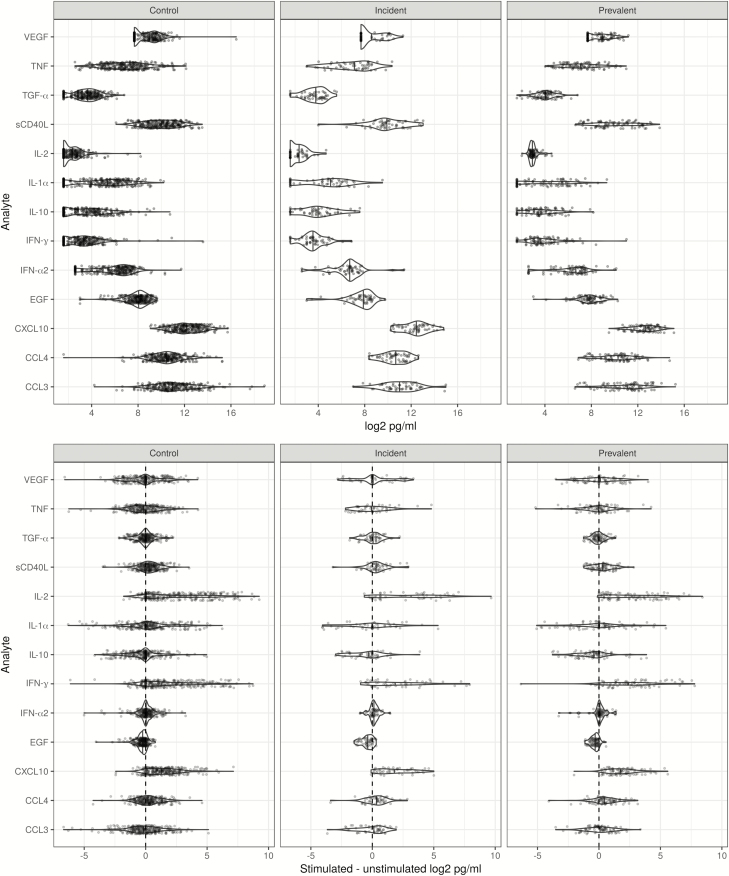
Raw data dot plots for unstimulated (top row) and antigen-stimulated, background-adjusted (bottom row) analytes in control, incident tuberculosis, and prevalent tuberculosis comparison groups. Distributions were estimated by violin plots with the median value indicated by a solid vertical line. All values are plotted on a log2 scale, and a vertical dashed line is plotted at a difference of 0. Abbreviations: CCL, chemokine (C-C motif) ligand; CXCL10, C-X-C motif chemokine 10; EGF, epidermal growth factor; IFN, interferon; IL, interleukin; sCD40L, soluble CD40 ligand; TGF-α, transforming growth factor-α; TNF, tumor necrosis factor; VEGF, vascular endothelial growth factor.

**Figure 2.**
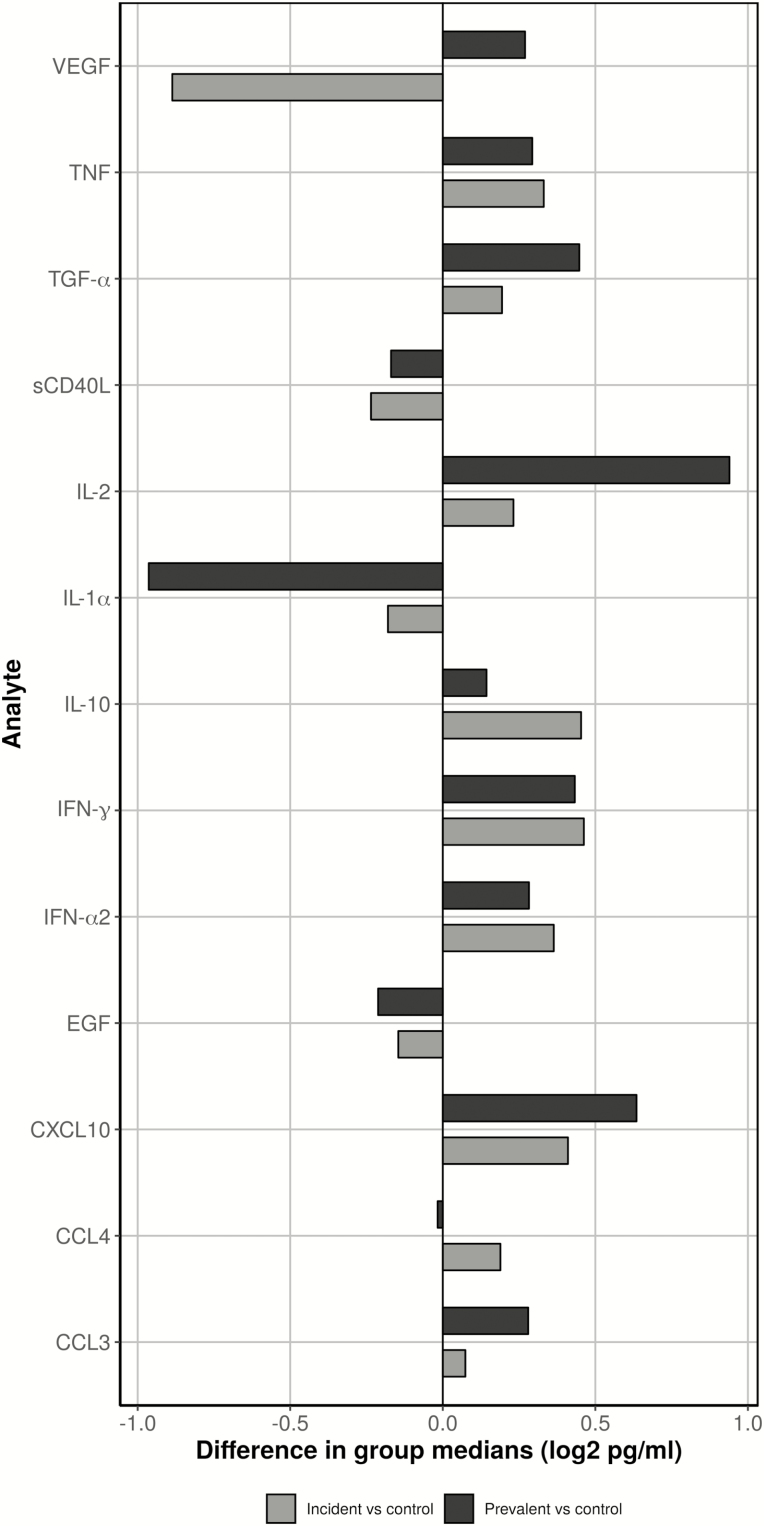
Standardized differences in unstimulated group medians (log2 pg/mL) between the incident tuberculosis group (grey) or prevalent tuberculosis group (black) vs the control group for each analyte. Negative differences mean the incident tuberculosis or prevalent tuberculosis group medians were lower than the control group medians. Abbreviations: CCL, chemokine (C-C motif) ligand; CXCL10, C-X-C motif chemokine 10; EGF, epidermal growth factor; IFN, interferon; IL, interleukin; sCD40L, soluble CD40 ligand; TGF-α, transforming growth factor-α; TNF, tumor necrosis factor; VEGF, vascular endothelial growth factor.

Analyte concentrations from unstimulated samples were higher in the incident and prevalent TB groups compared to the control group (Table 2, Figure 1, and Supplementary Figure S1). CXCL10, IL-2, and TGF-alpha were highest in the prevalent TB group. CCL4, IFN-alpha2, IFN-gamma, IL-10, and TNF were highest in the incident TB group; although not markedly higher than values seen in the prevalent TB group, the trend may indicate underlying inflammatory processes leading to TB risk. EGF, IL-1α, and CD40L were highest in the control group. A comparison between the control and prevalent TB groups showed that the largest group differences (>50% median difference, Figure 2) were in analytes CXCL10 and IL-2 (highest in prevalent), IL-1α (highest in control), and VEGF (lowest in incident). A statistical comparison between groups indicated CXCL10, IFN-γ, IL-2, and TGF-α remained statistically significant, both after FDR correction (Table 3, *P* corrected < 0.05) when comparing the control and prevalent TB groups and with the addition of IFN-α2 when comparing the control and TB-combined groups. No analytes remained statistically significant when comparing the control and incident TB groups after FDR correction, and only IL-2 remained significant when comparing the prevalent TB with incident TB groups (Table 3).

**Table 2.** Summary Measures for Each Analyte for the Unstimulated and Background-adjusted Values

Analyte	Control	Incident TB	Prevalent TB	TB-combined
Unstimulated				
**CCL3**	1973.9 (1070.1, 4427.7)	2077.9 (1111.4, 4098.6)	2396.5 (869.0, 4133.5)	2291.0 (969.4, 4117.9)
**CCL4**	1411.1 (809.9, 2280.0)	1608.4 (959.3, 2774.7)	1394.7 (660.8, 2186.4)	1450.5 (735.9, 2428.5)
**CXCL10**	4325.9 (2593.1, 8577.5)	5748.5 (2925.8, 8621.0)	6717.0 (3981.0, 10413.3)	6126.6 (3742.1, 9437.5)
**EGF**	274.0 (178.8, 380.4)	247.7 (148.6, 344.4)	236.6 (169.9, 321.2)	245.9 (164.0, 332.8)
**IFN-α2**	82.0 (29.0, 127.9)	105.5 (73.3, 145.8)	99.7 (51.3, 149.3)	104.7 (60.7, 148.7)
**IFN-γ**	8.6 (3.0, 15.2)	11.8 (8.0, 16.4)	11.5 (8.2, 23.0)	11.7 (8.0, 21.0)
**IL-10**	10.2 (3.0, 23.5)	14.0 (6.9, 27.7)	11.3 (6.1, 21.4)	12.5 (6.2, 25.7)
**IL-1α**	32.3 (4.8, 83.2)	28.5 (4.5, 59.6)	16.6 (3.6, 48.2)	20.6 (3.6, 56.7)
**IL-2**	4.0 (3.0, 6.6)	4.7 (3.0, 6.3)	7.7 (6.6, 8.5)	7.1 (5.6, 8.1)
**sCD40L**	1046.5 (490.5, 2102.6)	889.1 (645.0, 2260.7)	930.5 (415.7, 2915.1)	898.0 (454.6, 2494.2)
**TGF-α**	12.3 (7.1, 19.4)	14.1 (8.6, 20.6)	16.8 (12.1, 22.7)	16.1 (10.3, 22.1)
**TNF**	113.8 (47.5, 258.0)	143.1 (58.7, 300.6)	139.4 (67.1, 257.1)	139.4 (61.4, 283.1)
**VEGF**	379.0 (205.0, 694.0)	205.0 (205.0, 665.8)	456.9 (205.0, 701.5)	369.3 (205.0, 691.4)
**Background-adjusted, Stimulated–unstimulated**				
**CCL3**	-132.1 (-1197.0, 655.4)	169.7 (-1162.3, 750.3)	44.0 (-851.8, 704.4)	89.2 (-1026.5, 719.1)
**CCL4**	145.6 (-335.3, 965.1)	404.2 (-212.1, 940.1)	322.4 (-23.1, 1315.7)	351.6 (-76.2, 1124.0)
**CXCL10**	6144.6 (1698.8, 15576.7)	13 327.6 (4228.4, 34 531.8)	16 242.8 (4853.8, 31 249.0)	14 756.5 (4570.4, 31 947.6)
**EGF**	-37.5 (-76.6, -3.0)	-41.0 (-89.5, -5.1)	-43.6 (-76.5, -15.3)	-42.6 (-82.7, -10.6)
**IFN-α2**	0.0 (-10.1, 15.6)	9.1 (0.0, 24.3)	6.3 (-2.3, 16.6)	6.5 (0.0, 20.5)
**IFN-γ**	11.0 (0.0, 74.0)	27.2 (1.6, 89.3)	53.0 (13.4, 192.7)	44.2 (10.6, 177.6)
**IL-10**	0.0 (-7.5, 1.5)	-2.0 (-8.8, 3.0)	-2.3 (-8.4, 0.0)	-2.2 (-8.5, 0.0)
**IL-1α**	1.8 (-17.6, 25.5)	0.0 (-18.4, 13.3)	0.0 (-15.3, 16.4)	0.0 (-17.2, 16.0)
**IL-2**	14.0 (1.3, 86.3)	26.2 (1.8, 140.4)	33.4 (5.8, 152.8)	33.2 (5.4, 143.9)
**sCD40L**	152.4 (-91.3, 970.7)	291.8 (-55.3, 724.6)	153.4 (-78.2, 1151.1)	170.7 (-65.1, 864.5)
**TGF-α**	0.0 (-3.6, 2.4)	0.0 (-3.0, 4.0)	-0.6 (-4.4, 2.6)	0.0 (-4.1, 3.4)
**TNF**	-7.8 (-74.7, 39.3)	0.7 (-104.2, 35.0)	-1.0 (-83.6, 49.9)	-0.2 (-92.9, 40.3)
**VEGF**	0.0 (-227.8, 200.9)	0.0 (-116.7, 298.3)	0.0 (-239.0, 370.7)	0.0 (-197.5, 341.2)

The TB-combined group includes both the TB incident and TB prevalent groups. Values are shown as pg/ml median (IQR).

Abbreviations: CCL, chemokine (C-C motif) ligand; CXCL10, C-X-C motif chemokine 10; EGF, epidermal growth factor; IFN, interferon; IL, interleukin; IQR, interquartile range; sCD40L, soluble CD40 ligand; TB, tuberculosis; TGF-α, transforming growth factor-α; TNF, tumor necrosis factor; VEGF, vascular endothelial growth factor.

**Table 3.** *P* Values for Group Comparisons Using the Unstimulated Values and the Background-adjusted Values

	Control vs Combined		Control vs Culture-positive		Control vs Incident		Control vs Prevalent		Control vs Smear-negative		Prevalent vs Incident	
	Raw *P* Value	Adjusted *P* Value	Raw *P* Value	Adjusted *P* Value	Raw *P* Value	Adjusted *P* Value	Raw *P* Value	Adjusted *P* Value	Raw *P* Value	Adjusted *P* Value	Raw *P* Value	Adjusted *P* Value
Unstimulated												
**CCL3**	.7950	.9154	.9996	.9996	.9385	.9996	.7701	.9154	.8610	.9733	.8947	.9970
**CCL4**	.7551	.9154	.5566	.7616	.3007	.5097	.2410	.4272	.3624	.5543	.1055	.2351
**CXCL10**	.0082	**.0375**	.0051	**.0251**	.4181	.5947	.0030	**.0170**	.0138	.0569	.1701	.3266
**EGF**	.0872	.2000	.1597	.3193	.1416	.2907	.2196	.3984	.1752	.3266	.6930	.8718
**IFN-α2**	.0047	**.0246**	.0360	.1124	.0148	.0576	.0434	.1249	.0662	.1564	.5752	.7736
**IFN-γ**	<.0001	**.0002**	<.0001	**.0004**	.0138	.0569	.0001	**.0005**	.0005	**.0031**	.4194	.5947
**IL-10**	.0299	.0973	.0654	.1564	.0511	.1375	.1327	.2875	.1375	.2899	.3347	.5221
**IL-1α**	.0257	.0870	.0255	.0870	.3773	.5582	.0186	.0690	.0446	.1249	.2997	.5097
**IL-2**	<.0001	**<.0001**	<.0001	**<.0001**	.9980	.9996	<.0001	**<.0001**	<.0001	**<.0001**	<.0001	**<.0001**
**sCD40L**	.9480	.9996	.9733	.9996	.9091	.9987	.9968	.9996	.7203	.8918	.7947	.9154
**TGF-α**	.0004	**.0027**	.0001	**.0008**	.3256	.5183	.0001	**.0004**	.0004	**.0027**	.0575	.1496
**TNF**	.3179	.5166	.1759	.3266	.6603	.8584	.3071	.5097	.3793	.5582	.7981	.9154
**VEGF**	.4774	.6649	.6546	.8584	.0606	.1525	.6888	.8718	.9970	.9996	.0448	.1249
**Background-adjusted, Stimulated-unstimulated**												
**CCL3**	.3146	.5707	.6599	.7993	.5153	.7303	.3806	.6316	.6651	.7993	.9683	.9802
**CCL4**	.0540	.2004	.0486	.1994	.4163	.6494	.0459	.1994	.0479	.1994	.6528	.7993
**CXCL10**	<.0001	**<.0001**	<.0001	**<.0001**	.0024	**.0145**	<.0001	**<.0001**	<.0001	**<.0001**	.4480	.6721
**EGF**	.1745	.4002	.1485	.3862	.4281	.6548	.2124	.4555	.1538	.3869	.8496	.9202
**IFN-α2**	.0101	.0564	.0516	.2004	.0129	.0672	.1000	.2890	.1349	.3629	.2451	.4878
**IFN-γ**	<.0001	**.0004**	<.0001	**<.0001**	.1873	.4175	<.0001	**.0001**	<.0001	**<.0001**	.0722	.2347
**IL-10**	.1731	.4002	.2318	.4758	.7167	.8344	.1171	.3262	.0691	.2345	.4862	.7023
**IL-1α**	.2502	.4878	.3897	.6332	.4145	.6494	.3421	.5929	.1743	.4002	.9067	.9430
**IL-2**	.0020	**.0132**	.0011	**.0083**	.2161	.4555	.0012	**.0083**	0.0004	.0038	.2606	.4958
**sCD40L**	.4807	.7023	.9802	.9802	.6365	.7993	.5466	.7336	.8192	.9202	.9437	.9686
**TGF-α**	.8734	.9206	.8524	.9202	.3745	.6316	.6661	.7993	.6297	.7993	.3338	.5918
**TNF**	.7048	.8329	.5549	.7336	.8612	.9202	.5243	.7303	.8372	.9202	.5340	.7307
**VEGF**	.0668	.2345	.0176	.0859	.2712	.5036	.0970	.2890	.0907	.2831	.7961	.9132

*P* values remaining significant after false-discovery rate adjustment are in bold.

Abbreviations: CCL, chemokine (C-C motif) ligand; CXCL10, C-X-C motif chemokine 10; EGF, epidermal growth factor; IFN, interferon; IL, interleukin; sCD40L, soluble CD40 ligand; TGF-α, transforming growth factor-α; TNF, tumor necrosis factor; VEGF, vascular endothelial growth factor.

### Analytes From Stimulated Samples Corrected for Background

A number of analytes had higher unstimulated concentrations, so background correction could result in negative median (Ag-nil) differences (most notably TNF, IL-1α, IL-10, EGF, and CCL3; Table 2, Table 3, and Supplementary Figure S2). In the control vs prevalent TB and control vs TB-combined group comparisons, CXCL10, IFN-γ, and IL-2 remained statistically significant after FDR correction. CXCL10 also remained significantly different in the control vs incident TB group comparison; however, no analyte remained significant for (Ag-Nil) after FDR correction in the prevalent vs incident TB group comparison.

### Weighted Correlation Network Analysis

**Figure 3.**
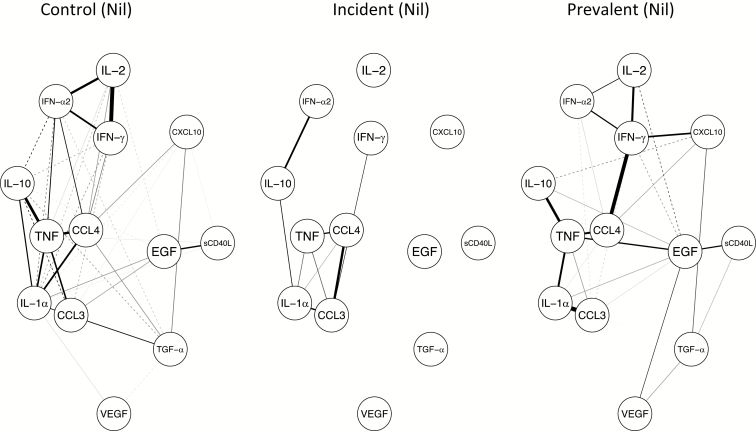
Weighted correlation networks in (left to right) controls, incident, and prevalent tuberculosis groups. Solid lines are indicative of positive correlations, dashed lines indicate negative correlations, and the strength of a correlation is indicated by the thickness of the line. Abbreviations: CCL, chemokine (C-C motif) ligand; CXCL10, C-X-C motif chemokine 10; EGF, epidermal growth factor; IFN, interferon; IL, interleukin; sCD40L, soluble CD40 ligand; TGF-α, transforming growth factor-α; TNF, tumor necrosis factor; VEGF, vascular endothelial growth factor.

A weighted correlation network analysis on the nil analyte concentrations (Figure 3) demonstrated a cluster of positively-correlated analytes, including IL-2, IFN-γ, and INF-α2 in the control group and, to a lesser extent, in the prevalent TB group, which is absent in the incident TB group. The second cluster identified included CCL3, CCL4, IL-1α, IL-10, and TNF, with varying strengths. While this cluster was present in all 3 groups, it is notable that the correlation between CCL3 and CCL4 was positive only in the incident TB group (Figure 3). The same analysis on the background-corrected analyte values led to a similar identification of an IL-2 and IFN-γ correlation, but this time in association with CXCL10 instead of IFN-α2 (Supplementary Figure S3). This cluster was present in all 3 groups. There was a strong correlation, in the incident TB group only, between INF-γ and INF-α2, which was small and negative in both the control and prevalent TB groups. There were notable negative correlations in the TB incident group, specifically between IL-10 and INF-γ, while a strong positive correlation was seen between IFN-γ and IFN- α2, but was present in this group only.

### Predictive Models

**Figure 4.**
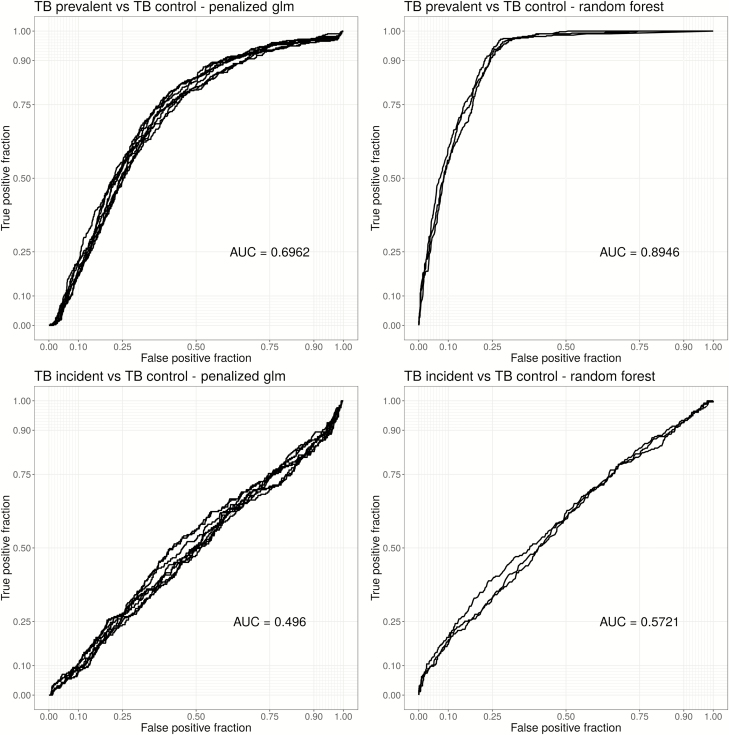
Receiver operator curves (ROCs) for primary comparison using nil data. Each curve represents an independent, cross-validated, predictive model with different tuning parameters. The left panels are curves estimated using glmnet-penalized regression and the right panel ROC curves were estimated using random forests: each row corresponds to a specific comparison. Abbreviations: AUC, area under the curve; TB, tuberculosis.

Results of predictive model fitting showed the tree-based ensemble models of random forests to be most accurate, although more interpretable models (glmnet) also performed reasonably well by comparison with AUC (Figure 4, Supplementary Table S5, and Supplementary Figure S4). Models using the unstimulated analyte values as input were better able to discriminate between the control and prevalent TB groups (random forest AUC = 0.9, glmnet AUC = 0.7) than models using the background-corrected (Ag-Nil) values. The discrimination between the incident TB and prevalent TB groups was also reasonable (AUC > 0.8); however, the models poorly discriminated between the control and incident TB groups. Model-predicted variable importance scores ranked IL-2 and IFN-γ at or near the top for all comparisons, except the comparison between the control vs incident TB group (Table 4). Models using the Ag-Nil values as the input performed poorly by comparison, requiring a large number of analytes to reach an overall lower AUC (max AUC < 0.7 for all models; Table 4).

**Table 4.** Analyte and Variable Importance Scores (Scaled) Estimated From Elastic Net Penalization Are Reported in Order of Importance for Each Comparison Using Unstimulated and Background-adjusted Values

Control vs Incident	Control vs Prevalent	Prevalent vs Incident	Control vs Combined
Unstimulated			
**CCL3 (100)**	**IFN-γ (100)**	**IL-2 (100)**	**IFN-γ (100)**
**CCL4 (26)**	**IL-2 (78)**	**EGF (28)**	**IL-2 (56)**
**IFN-α2 (20)**	TNF (19)	IFN-γ (10)	**IFN-α2 (38)**
TNF (16)	CCL3 (18)	CCL4 (9)	CCL4 (13)
IL-10 (12)	IFN-α2 (11)	TGF-α (6)	TGF-α (12)
IL-2 (8)	CXCL10 (7)	sCD40L (1)	IL-1α (6)
EGF (6)	IL-1α (6)	TNF (1)	EGF (5)
VEGF (5)	IL-10 (5)		VEGF (4)
IFN-γ (4)	sCD40L (5)		sCD40L (3)
sCD40L (4)	VEGF (2)		IL-10 (2)
CXCL10 (1)	TGF-α (2)		CCL3(1)
IL-1α (1)	EGF (1)		TNF (1)
**Background-adjusted, Stimulated-unstimulated**			
**CCL3 (100)**	**CXCL10 (100)**	**IFN-α2 (100)**	**IFN-γ (100)**
**CXCL10 (57)**	**CCL4 (59)**	**CCL4 (59)**	**TNF (77)**
**IL-2 (52)**	**IL-2 (58)**	**sCD40L (53)**	**IFN-α2 (62)**
**IFN-γ (47)**	**TGF-α (35)**	**IFN-γ (40)**	**EGF (52)**
**TNF (40)**	**sCD40L (31)**	**IL-10 (38)**	**IL-1α (46)**
**CCL4 (34)**	**VEGF (20)**	VEGF (14)	**IL-10 (38)**
**IL-1α (32)**	**IFN-γ (20)**	EGF (11)	**VEGF (36)**
**IFN-α2 (26)**	CCL3 (19)	IL-2 (11)	**CXCL10 (33)**
VEGF (19)	TNF (14)	TGF-α (10)	**sCD40L (32)**
EGF (11)	IL-1α (14)	IL-1α (9)	**CCL4 (28)**
sCD40L (5)	IFN-α2 (12)	CXCL10 (1)	TGF-α (18)
IL-10 (1)	IL-10 (3)		IL-2 (3)

Analytes that do not appear had a variable importance score of 0.

Bold items are analytes that were found to be significant for prediction >20% of the time.

Abbreviations: CXCL10, C-X-C motif chemokine 10; CCL, chemokine (C-C motif) ligand; EGF, epidermal growth factor; IFN, interferon; IL, interleukin; sCD40L, soluble CD40 ligand; TGF-α, transforming growth factor-α; TNF, tumor necrosis factor; VEGF, vascular endothelial growth factor.

### Sensitivity Analyses

There were 4 additional sensitivity analyses. First, we utilized the culture-confirmed TB samples (n = 106 from the 87 prevalent and 19 incident TB patients) in place of the TB-combined group as a comparison group (Table 1). After FDR adjustment, CXCL10, IFN-γ, IL-2, and TGF-α remained statistically significant in the control vs culture-confirmed comparison of the unstimulated values, while only CXCL10, IFN-γ, and IL-2 did so using the Ag-Nil values (Table 3). Second, we utilized the subset (n = 181) of individuals randomized to placebo (ie, those that did not receive IPT treatment) to investigate the development of incident TB (Supplementary Table S3). Baseline characteristics in this subgroup were comparable. Out of 334 randomized individuals, 181 (54%) were randomized to placebo; 33 of the 181 (18%) placebo recipients developed incident TB, compared to 18 of the 153 (12%) participants who were randomized to IPT treatment (95% confidence interval for difference, -0.01–0.12; *P* = .1016). No analytes remained significant after FDR correction. Third, the subset of sputum smear–negative TB-prevalent patients (n = 79, Tables 1 and 3) were compared to the controls. After FDR correction, IFN-γ, IL-2, and TGF-α remained statistically significant using the nil analyte values, and CXCL10 and IFN-γ remained significant when using the Ag-nil values. Fourth, based on TB diagnosis time in the incident TB group, split by median time to TB (<=61 weeks, >62 weeks; analyte values are summarized in Supplementary Table S4).

## DISCUSSION

We evaluated the predictive value of selected analytes as potential biomarkers for HIV-associated TB. This is the first study of this size, nested within a 4-year prospective cohort, using samples collected at the screening stage of a large RCT, that showed that adding IPT to ART reduced the TB incidence in patients with HIV-1 infections [8]. A unique feature of our study is the comparison of 13 biomarkers in the plasma from prevalent and incident TB patients. Novel TB biomarkers, other than IFN-γ, might significantly contribute to the prediction of prevalent TB and improve the multivariate risk prediction for incident TB.

Zak et al [12] and Suliman et al [13] successfully identified 6 and 4 gene RNA signatures, respectively, in peripheral blood that can partially predict a progression to TB. While these findings are important, a more applicable predictive tool would be a test based on measuring soluble markers in easily-accessible samples suited for use in primary or secondary healthcare facilities by health care personnel with minimal training.

Recent work has demonstrated that Quantiferon supernatants can be useful in identifying combination biomarkers to diagnose pulmonary TB [14, 15]. Our findings are in keeping with those described by Chegou et al, although with some differences, as their patient population was HIV-uninfected [16]. Significantly different analyte concentrations between groups in the unstimulated samples were seen in CXCL10, IFN-γ, IL-2, and TGF-α, when comparing the control with prevalent TB groups, and also with IFN-α2 when comparing the control with TB-combined groups. The role of CXCL10, with or without IFN-γ, in both adult and paediatric populations, has been highlighted previously [17, 18]. In our analysis, CXCL10 remained statistically significant between both the control and prevalent groups, as well as the incident TB group, after correction for multiple comparisons in background-corrected, antigen-stimulated samples. The finding that unstimulated IL-2 concentrations differentiated prevalent from incident TB samples after correction for statistical comparisons suggests that IL-2 may be an important marker of TB progression and warrants further investigation, although the median concentrations are low. Our predictive model analysis also ranked unstimulated IL-2 (together with IFN-γ) highly for all comparisons, except for control versus incident TB. There is a dynamic relationship between IFN-γ– and IL-2–secreting antigen-specific T cells in patients during and after treatment for TB, reflecting that it is likely driven by antigen load [19]. These findings illustrate that the ability of unstimulated plasma analyte concentrations to better identify TB risks in these HIV-1–infected patients demonstrates underlying inflammatory processes, and higher overall background activation might render them more susceptible to progression to TB.

While our study has strengths, in particular of power, it also has limitations, including the limited number of preselected analytes measured, the lack of an external validation cohort, and the sampling, which relied on sample availability, leading to possible bias (ie, the lack of QFT-indeterminate individuals in the analysis set). The randomization to IPT or placebo also affected the outcome of our investigation, since IPT reduced incident TB, also reducing the ability of biomarkers to predict it in this cohort. Additionally, ongoing TB exposure post-enrollment could have contributed to the inability of these markers to predict incident TB, potentially measuring the baseline plasma biomarkers that were present prior to the TB exposure and that may have led to active disease. Additional weaknesses include poor predictive performance in the analysis and unbalanced classes, since down-sampling reduces the effective sample size. Classification performance may also be impacted by unmeasured confounding and effect modification, potentially related to the unique setting in South Africa. However, with these limitations in mind, our data, indicating that a combination of plasma biomarkers may be used to detect prevalent TB in HIV-1–infected individuals, is potentially an important finding. It is also interesting that unstimulated analytes performed best, therefore potentially avoiding the need to culture cells or whole blood in vitro. Our findings could pave the way towards further, larger, prospective studies that would evaluate soluble plasma biomarkers that might effectively predict incident TB in HIV-infected patients.

## Supplementary Data

Supplementary materials are available at *Clinical Infectious Diseases* online. Consisting of data provided by the authors to benefit the reader, the posted materials are not copyedited and are the sole responsibility of the authors, so questions or comments should be addressed to the corresponding author.

ciy823_suppl_Supplement-Material

## Notes

***Author contributions.*** M. X. R., R. J. W., and K. A. W. were involved in the conception and design. M. X. R., G. M., R. G., S. M., J. M.-K., R. J. W., and K. A. W. were involved in the study implementation. M. L. and C. P. did the analysis, with early input from A. K. C., K. A. W., and M. X. R. The data were interpreted by M. L., K. A. W., and R. J. W., who also provided important intellectual input and wrote the first draft. All authors read and contributed to the final manuscript. K. A. W. had full access to all the data in the study and had final responsibility for the decision to submit for publication.

***Acknowledgments.*** The authors thank the individuals and staff at the Ubuntu clinic in Khayelitsha, South Africa. They also thank the Provincial Government of the Western Cape and City of Cape Town Health Department for integrating the antiretroviral therapy–isoniazid preventive therapy study into the clinic data and patient systems. They thank the Treatment Action Campaign of Khayelitsha for providing invaluable waiting-room patient literacy and advocacy.

***Disclaimer.*** The funding sources had no role in the study design, data collection, data analysis, data interpretation, or writing of the manuscript.

***Financial support.*** This work was supported by the Department of Health of South Africa, Wellcome Trust (grant numbers 084670, 104803, and 203135), the Foundation for Innovation and New Diagnostics, the European Union Horizon 2020 research and innovation programme (grant agreement number 643381), and Hasso Plattner (Institute of Infectious Diseases and Molecular Medicine, University of Cape Town).

***Potential conflicts of interest.*** M. X. R. is supported by a Clinical Excellence Fellowship award administered by University College London. R. J. W. and K. A. W. are supported by the Francis Crick Institute, which receives its core funding from Cancer Research UK (grant number FC001218), the UK Medical Research Council (grant number FC001218), and Wellcome (grant number FC001218). R. J. W. also receives support from the National Institutes of Health (grant numberUI9 AI111276); the Medical Research Council of South Africa, via it’s strategic health innovations partnership; and from the National Research Foundation of South Africa (grant number 96841). All other authors report no potential conflicts. All authors have submitted the ICMJE Form for Disclosure of Potential Conflicts of Interest. Conflicts that the editors consider relevant to the content of the manuscript have been disclosed.
