# Supplementary material for: Plasma Biomarkers to Detect Prevalent or Predict Progressive Tuberculosis Associated With Human Immunodeficiency Virus–1
Source: Clin Infect Dis. 2018 Sep 26;69(2):295–305. doi: 10.1093/cid/ciy823 (PMC6603269; doi:10.1093/cid/ciy823)
Supplement: ciy823_suppl_Supplement-Material [file ciy823_suppl_supplement-material.pdf]

Supplementary appendix to:

Plasma biomarkers to detect prevalent, or predict progressive, HIV-1-associated tuberculosis

Maia Lesosky[1,2]

Molebogeng X Rangaka[2,3]

Cara Pienaar[1]

Anna K Coussens[2,4]

Rene Goliath[2]

Shaheed Mathee[5]

Judith Mwansa-Kambafwile[2]

Gary Maartens[3]

Robert J Wilkinson[2,3,6,7]

Katalin A Wilkinson[2,3,7*]

1. Division of Epidemiology & Biostatistics, School of Public Health and Family Medicine, University of Cape Town, Observatory 7925, Republic of South Africa
2. Wellcome Centre for Infectious Diseases Research in Africa, Institute of Infectious Diseases and Molecular Medicine, University of Cape Town, Observatory 7925, Republic of South Africa
3. Department of Medicine, Faculty of Health Sciences, University of Cape Town, Observatory 7925, Republic of South Africa
4. Department of Pathology, Faculty of Health Sciences, University of Cape Town, Observatory 7925, Republic of South Africa
5. Site B Khayelitsha Community Health Centre, Western Cape Department of Health, Khayelitsha, Republic of South Africa
6. Department of Medicine, Imperial College London, Norfolk Place, London W2 1PG, UK
7. The Francis Crick Institute, 1 Midland Road, London, NW1 2AT

* To whom correspondence should be directed at the Francis Crick Institute: Katalin.Wilkinson@crick.ac.uk, tel +44 203 796 2304

Supplement detailed methods

Sampling
From the 2173 individuals screened as part of the parent study, a subset were willing to provide additional consent for further screening by IGRA and TST. Samples in this analysis included all individuals with an IGRA sample available from the parent study, who had either prevalent or incident TB, as well as two controls for each prevalent and incident case, selected randomly based on IGRA availability, in the order of recruitment to be just before and just after the recruitment of the prevalent or incident case. The IGRA samples were taken as part of a sub-study nested within the parent population as described in Rangaka et al "Interferon release does not add discriminatory value to smear-negative HIV-tuberculosis algorithms" in the European Respiratory Journal 2012, 39:163-171.

Standard curves
Analyte concentrations were calculated with reference to the standard curve for each analyte, ranging from 3.2 pg/ml to 10000 pg/ml in the Milliplex assay, according to the manufacturer's instructions. Manufacturer supplied internal controls (QC1 and QC2) were used to validate the standard curves. The minimal detection limit was additionally guided by the observed/expected values of the standard concentrations being between (70-130) %. Next, the corresponding (fluorescence-background) results were checked to ensure they are >0, thus further ensuring that we detect meaningful concentrations. The mean lower limit of detection for each analyte was calculated using data from the 11 plates used to run all samples. These values are given in the methods section in pg/ml, and they were consistent at both the lower and top end of the standard curve, except for VEGF, which had a sensitivity of 205 pg/ml, calculated as above. Raw out of range (OOR) values were adjusted, by replacing all OOR< (lower than the lowest detectable value) with 0 (1027 instances), and replacing all >OOR (higher than the highest detectable value) with 10000 pg/mL (top standard) (13 instances) and adding the mean limit of detection (LOD) per analyte to all plates.

Statistical methods
Results are presented for unstimulated (Nil) and stimulated – nil (TB Ag-Nil) values. Analyte values are nearly always presented in log2 transformed scale as log2 pg/ml. Subgroups for analysis included incident TB, prevalent TB, controls and the combined incident and prevalent TB groups, hereafter referred to as TB-combined. Frequency (percent) or median (inter-quartile range) were calculated by group for discrete and continuous values respectively. Sensitivity analyses were undertaken using the subgroup of culture-confirmed incident TB, the subgroup randomised to placebo, and the subgroup of prevalent TB who were smear negative at baseline (Smear-negative). Demographic and clinical characteristics were also calculated for entire screening population of the parent study, from whom the samples in this analysis were drawn. Time to onset of TB was calculated as the days between date of screening and first date of TB registration.

Statistical tests to compare groups were Fisher's exact test or Wilcoxon rank sum test, as appropriate. Throughout, a nominal threshold for statistical significance was set at $\alpha = 0{\cdot}05$, and false discovery rate correction (FDR) by Benjamini-Hochberg[9] was applied. These values are reported as p-corrected. Data visualisation was used to clarify differences between and within groups.

Weighted correlation network analysis was carried out on the nil and background corrected analyte levels, stratified by TB status and presented with correlation diagrams using the package *qgraph* (Epskamp et al, 2012). Correlation matrixes were first estimated using Pearson's correlation of the $\log_2$ transformed data in the case of the nil values and z-score transformed data in the case of the Ag-nil data (due to the presence of negative values). The partial correlation network was estimated using the *glasso* internal method with tuning parameter gamma set to = 0.15. All other settings remained at default. Thicker edges indicate stronger associations, positive associations are represented with solid lines while negative associations are represented by dashed lines.

Supervised learning models were applied to the data to predict class membership (e.g. incident vs prevalent TB) in 2-way classifications using the *caret* (Kuhn et al, 2018) package. In all cases analyte values were centred and scaled prior to input and all models were carried out with 10-fold cross-validation resampling to estimate classification accuracy. Cross-validation is a form of internal validation that generates independent 'training' and 'evaluation' samples from the same data source, whereby the learning models are applied multiple times and a pooled estimate of final classification accuracy obtained. Sampling was stratified by down sampling to ensure balanced class representation in the re-samples. Classification learners assessed included random forests (RF) to set a performance ceiling (Liaw and Weiner, 2002) and elastic-net regularisation (glmnet) (Friedman, Hastie, Tibshirani, 2010) for a potentially interpretable and applicable model. Elastic-net regularisation is a type of penalised regression that results in covariate selection by down-weighting covariates that do not contribute to classification accuracy.

Models were optimised by evaluation of area under the curve (AUC) of the receiver operator characteristic (ROC), and this metric along with ranges reported. Models were selected on the basis of largest minimum unbiased AUC estimate. Cross-validated AUC and ranges were estimated. Receiver-operator curves were generated using the predicted vs observed classifications for each independent model and were drawn for all models as there were only minor differences in performance across different model parameters. Training for the prediction models utilising the default grid search approach for the parameters with a specified grid length. For the random forest analyses, the grid searched over the parameter *mtry* which controls the number of possible samples at each node. Penalised regression searched for optimal values over the two parameters alpha and lambda, which adjust the elastic net penalisation weights.

Variable importance score (VI), calculated as a scaled beta coefficient, was used as the primary means of determining individual analyte impact on classification outcome. Lists of analytes and associated VI score are presented. All statistical analysis was carried out in R v.3.3 (R Core Team).

Table S1

Analytes assayed in this study and the reason for their selection.

**Analyte [abbreviation] (limit of detection)**	**Reason**	**Reference**
Interferon-γ [IFN-γ] (3)	A widely researched correlate of MTB sensitisation and T-cell effector function	O'Garra, Redford, McNab et al. (2013) *Annu Rev Immunol.*
Interferon-α2 [IFN-α2] (6)	Type I IFN responses are associated with active TB	Berry, Graham, McNab et al. (2010) Nature.
Interleukin-2 [IL-2] (3)	Essential for T cell proliferation and generation of a T cell memory response during antigen recognition, secreted by polyfunctional T cells	Beveridge, Price, Casazza et al. (2007) *Eur J Immunol.*
CXCL-10 [IP-10] (10)	Type II interferon inducible T cell attracting chemokine, shown to be a useful biomarker for differentiating patients from controls	Ruhwald, Bjerregaard-Anderson, Rabna et al. (2007) *Microbes Infect.*<br>Ruhwald, Dominguez, Latorre et al. (2011) *Tuberculosis*
Interleukin-10 [IL-10] (3)	A regulatory cytokine, induced by BCG vaccination in newborns	Guyot-Revol, Innes, Hackforth et al. (2006) *AJRCCM*<br>Hanekom (2005) *Ann NY Acad Sci.*
Vascular endothelial growth factor [VEGF] (205)	Shown to differentiate active TB from controls in combination with EGF, sCD40L, IL-1 TGF-α	Chegou, Black, Kidd et al. (2009) *BMC Pulm Med*.
Epidermal growth factor [EGF] (8)	As above	Chegou, Black, Kidd et al. (2009) *BMC Pulm Med*.
Soluble CD40 Ligand [sCD40L] (16)	As above	Chegou, Black, Kidd et al. (2009) *BMC Pulm Med*.
Tumor growth factor α TGF-α (3)	As above	Chegou, Black, Kidd et al. (2009) *BMC Pulm Med*.
Interkeukin-1α [IL-1α] (3)	As above	Chegou, Black, Kidd et al. (2009) *BMC Pulm Med*.
Macrophage inflammatory Protein 1α /Combined chemokine ligand 3, CCL-3 [MIP-1α] (7)	As above	Chegou, Black, Kidd et al. (2009) *BMC Pulm Med*.
MIP-1β /CCL-4 (3) [MIP-1β] (3)	As above	Chegou, Black, Kidd et al. (2009) *BMC Pulm Med*.
Tumour Necrosis Factor [TNF-α] (3)	Promising diagnostic marker for active TB	Harari , Rozot, Bellutti et al. (2011) *Nature Medicine*

Table S2: Demographic and clinical characteristics of all screened individuals from the parent study, and the sensitivity analysis subgroups of control and incident TB who were randomised to placebo with the overall analysis cohort for comparison. All values given as frequency (%) unless otherwise indicated.

Variable	Level	Manuscript main analysis n = 421	All screened (parent study) n = 2137	Control group (randomised to placebo) n = 148	Incident TB (randomised to placebo) n = 33
**BMI** (median, IQR)		25 (22, 30)	25 (22, 29)	27 (24, 31)	24 (21, 28)
**AGE** (years) (median, IQR)		34 (30, 39)	34 (29, 40)	34 (30, 38)	32 (30, 37)
**Sex**	**Male**	106 (25)	597 (28)	33 (22)	10 (30)
	**Female**	315 (75)	1539 (72)	115 (78)	23 (70)
**Prior TB**	**No**	234 (56)	1248 (58)	83 (56)	16 (50)
	**Yes**	184 (44)	827 (39)	65 (44)	16 (50)
	**Unknown**	3 (1)	60 (3)	-	-
**TST positive* (5mm)**	**No**	215 (56)	1421 (67)	82 (59)	18 (62)
	**Yes**	172 (44)	715 (33)	56 (41)	11 (38)
**TST positive* (10mm)**	**No**	225 (58)	1324 (62)	86 (62)	18 (62)
	**Yes**	162 (42)	812 (38)	52 (38)	11 (38)
**TB symptoms at baseline**	**No**	360 (86)	137 (93)	137 (93)	27 (82)
	**Yes**	61 (14)	11 (7)	11 (7)	6 (18)
**CD4 category**	**< 200**	196 (47)		59 (40)	16 (48)
	**200 - 350**	114 (27)		43 (29)	9 (27)
	**350 +**	110 (26)		46 (31)	8 (24)
**Ever ART**	**No**	169 (43)		50 (37)	11 (36)
	**Yes**	221 (57)		86 (63)	20 (64)
**ART < 3 months at baseline if ART**	**No**	196/221(92)		78/86 (91)	20/20 (100)
	**Yes**	25/221 (12)		8/86 (9)	0 (0)
**QFT status at baseline**	**Negative**	212 (50)	696 (33)	81 (55)	17 (52)
	**Positive**	208 (50)	554 (26)	66 (45)	16 (48)
	**Indeterminate**	1 (0)	88 (4)	-	-
	**Not done**	-	799 (37)	-	-
**Culture confirmed TB**	**Negative**	315 (75)	1594 (75)	148 (100)	19 (58)
	**Positive**	106 (25)	197 (9)	0 (0)	14 (42)
	**Indeterminate/Discordant**	-	52 (2)	-	-
	**Unknown/No result**	-	294 (14)	-	-
**INH Treatment**	**No**	199 (47)		148 (100)	33 (100)
	**Yes**	162 (38)		-	-
	**Unknown/No result**	60 (14)		-	-

* 1 missing TST result

Table S3

Summary measures (median, IQR) and p-values (raw, false-discovery rate (FDR) adjusted) for the unstimulated (A) and antigen-stimulated background-adjusted values (B) in the placebo treated control and TB incident sensitivity analysis groups.

	Analyte	Control (randomised to placebo)	TB Incident (randomised to placebo)	Control vs incident TB	
A		n = 148	n = 33	Raw p-value	FDR adjusted p-value
	CCL3	1770 (954, 3820)	2477 (1123, 4408)	0.3889	0.6741
	CCL4	1311 (816, 2219)	1944 (967, 3091)	0.1250	0.3612
	CXCL10	4420 (2601, 9190)	6461 (2523, 9338)	0.4726	0.6826
	EGF	255 (171, 362)	248 (183, 354)	0.6956	0.8374
	IFN-α2	83 (24, 124)	106 (76, 131)	0.0629	0.2423
	IFN-γ	8 (3, 14)	11 (8, 17)	0.0412	0.2423
	IL-10	9 (3, 24)	14 (10, 26)	0.0430	0.2423
	IL-1α	28 (4, 77)	28 (3, 66)	0.7086	0.8374
	IL-2	4 (3, 7)	5 (3, 7)	0.8636	0.8856
	sCD40L	975 (486, 2108)	835 (492, 1689)	0.4210	0.6744
	TGF-α	12 (7, 20)	13 (8, 20)	0.8240	0.8856
	TNF	107 (44, 244)	155 (56, 313)	0.2585	0.5602
	VEGF	449 (205, 693)	205 (205, 748)	0.3837	0.6741
B					
	CCL3	-160 (-967, 576)	101 (-2223, 786)	0.8184	0.9167
	CCL4	118 (-359, 711)	404 (-305, 1115)	0.4221	0.6455
	CXCL10	6518 (1910, 14948)	10551 (3960, 32495)	0.0247	0.3157
	EGF	-34 (-73, 0)	-41 (-94, -10)	0.2648	0.5780
	IFN-α2	2 (-7, 15)	11 (0, 24)	0.0699	0.3552
	IFN-γ	9 (0, 60)	23 (2, 144)	0.3429	0.5944
	IL-10	0 (-8, 0)	0 (-10, 4)	0.8168	0.9167
	IL-1α	1 (-11, 24)	0 (-10, 15)	0.7334	0.9081
	IL-2	13 (1, 68)	19 (2, 110)	0.3112	0.5780
	sCD40L	155 (-91, 1010)	89 (-59, 621)	0.6553	0.8519
	TGF-α	0 (-3, 2)	1 (-3, 4)	0.0956	0.3552
	TNF	-14 (-76, 23)	-14 (-93, 34)	0.9897	0.9897
	VEGF	0 (-253, 108)	0 (-173, 338)	0.1685	0.4867

Table S4

Summary measures (median, IQR) for the unstimulated (A) and antigen-stimulated background-adjusted values (B) in the TB incident sensitivity analysis by time of TB diagnosis groups.

	Analyte	TB Incident (TB Dx in 0-61 weeks)	TB Incident (TB Dx in 62-177 weeks)
**A**		**n = 25**	**n = 26**
	**CCL3**	2078 (799, 3896)	1895 (1159, 5190)
	**CCL4**	1608 (735, 2705)	1602 (1163, 3040)
	**CXCL10**	4782 (2523, 6690)	6165 (4574, 10403)
	**EGF**	217 (146, 323)	256 (173, 353)
	**IFN-α2**	99 (76, 125)	110 (72, 163)
	**IFN-γ**	10 (8, 16)	12 (8, 17)
	**IL-10**	15 (10, 32)	13 (6 23)
	**IL-1α**	28 (3, 57)	31 (17, 83)
	**IL-2**	5 (3, 7)	5 (3, 6)
	**sCD40L**	907 (631, 2441)	867 (662, 1724)
	**TGF-α**	10 (7, 15)	18 (12, 22)
	**TNF**	143 (35, 292)	142 (74, 302)
	**VEGF**	205 (205, 696)	205 (205, 504)
**B**			
	**CCL3**	172 (-1119, 422)	103 (-1846, 785)
	**CCL4**	341 (-185, 964)	420 (-424, 704)
	**CXCL10**	10205 (2980, 32495)	13842 (6271, 34166)
	**EGF**	-62 (-102, -7)	-28 (-57, -3)
	**IFN-α2**	12 (0, 25)	8 (-5, 23)
	**IFN-γ**	17 (0, 75)	33 (6, 131)
	**IL-10**	0 (-9, 3)	-2 (-9, 2)
	**IL-1α**	0 (-19, 15)	0 (-17, 12)
	**IL-2**	13 (2, 106)	52 (3, 141)
	**sCD40L**	31 (-82, 758)	340 (38, 685)
	**TGF-α**	1 (-3, 3)	-1 (-4, 5)
	**TNF**	1 (-80, 48)	-6 (-119, 28)
	**VEGF**	0 (-16, 352)	0 (-145, 219)

Table S5

Mean, minimum and maximum AUC estimates generate for each independent run of the cross-validated predictive model, using nil analyte values and ag-nil analyte values.

Model	Mean AUC	Minimum AUC	Maximum AUC
Nil analyte values			
TB combined vs TB control - penalised glm	0.62	0.60	0.63
TB incident vs TB control - penalised glm	0.51	0.50	0.52
TB prevalent (culture confirmed) vs TB control - penalised glm	0.67	0.64	0.69
TB prevalent (smear negative) vs TB control - penalised glm	0.72	0.71	0.73
TB prevalent vs TB control - penalised glm	0.72	0.72	0.72
TB prevalent vs TB incident - penalised glm	0.74	0.71	0.81
TB combined vs TB control - random forest	0.76	0.76	0.77
TB incident vs TB control - random forest	0.58	0.57	0.59
TB prevalent (culture confirmed) vs TB control - random forest	0.82	0.81	0.82
TB prevalent (smear negative) vs TB control - random forest	0.90	0.89	0.90
TB prevalent vs TB control - random forest	0.89	0.89	0.89
TB prevalent vs TB incdient - random forest	0.86	0.86	0.87
Ag-nil analyte values			
TB combined vs TB control - penalised glm	0.61	0.60	0.62
TB incident vs TB control - penalised glm	0.58	0.57	0.58
TB prevalent (culture confirmed) vs TB control - penalised glm	0.60	0.59	0.6
TB prevalent (smear negative) vs TB control - penalised glm	0.65	0.64	0.66
TB prevalent vs TB control - penalised glm	0.60	0.59	0.60
TB prevalent vs TB incident - penalised glm	0.58	0.56	0.61
TB combined vs TB control - random forest	0.67	0.67	0.67
TB incident vs TB control - random forest	0.55	0.53	0.56
TB prevalent (culture confirmed) vs TB control - random forest	0.66	0.66	0.66
TB prevalent (smear negative) vs TB control - random forest	0.66	0.64	0.67
TB prevalent vs TB control - random forest	0.70	0.69	0.70
TB prevalent vs TB incident - random forest	0.53	0.52	0.54

Figure S1

Selected analytes (CXCL10/IP-10, INF-gamma, IL-2, TGF-alpha) comparing Control, Incident TB, Prevalent TB and TB-combined for unstimulated values (log2 scale). Density estimated with violin plot and median value plotted as solid horizontal line.

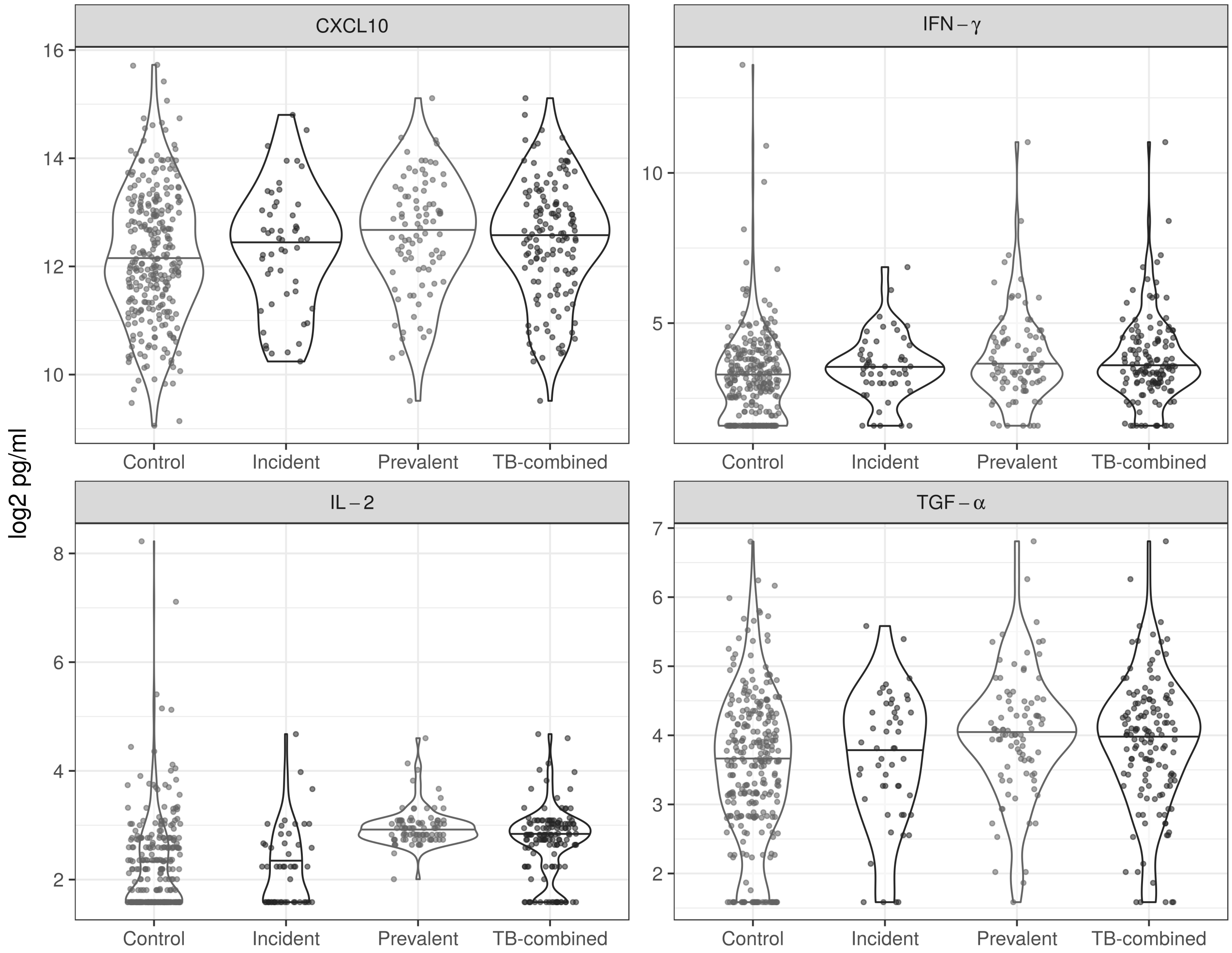

Figure S2

Selected analytes (4 mediators: CXCL10, INF-γ, IL-2, TGF-α) comparing Control, Incident TB, Prevalent TB and TB-combined for antigen-stimulated background-adjusted values (log2 scale).

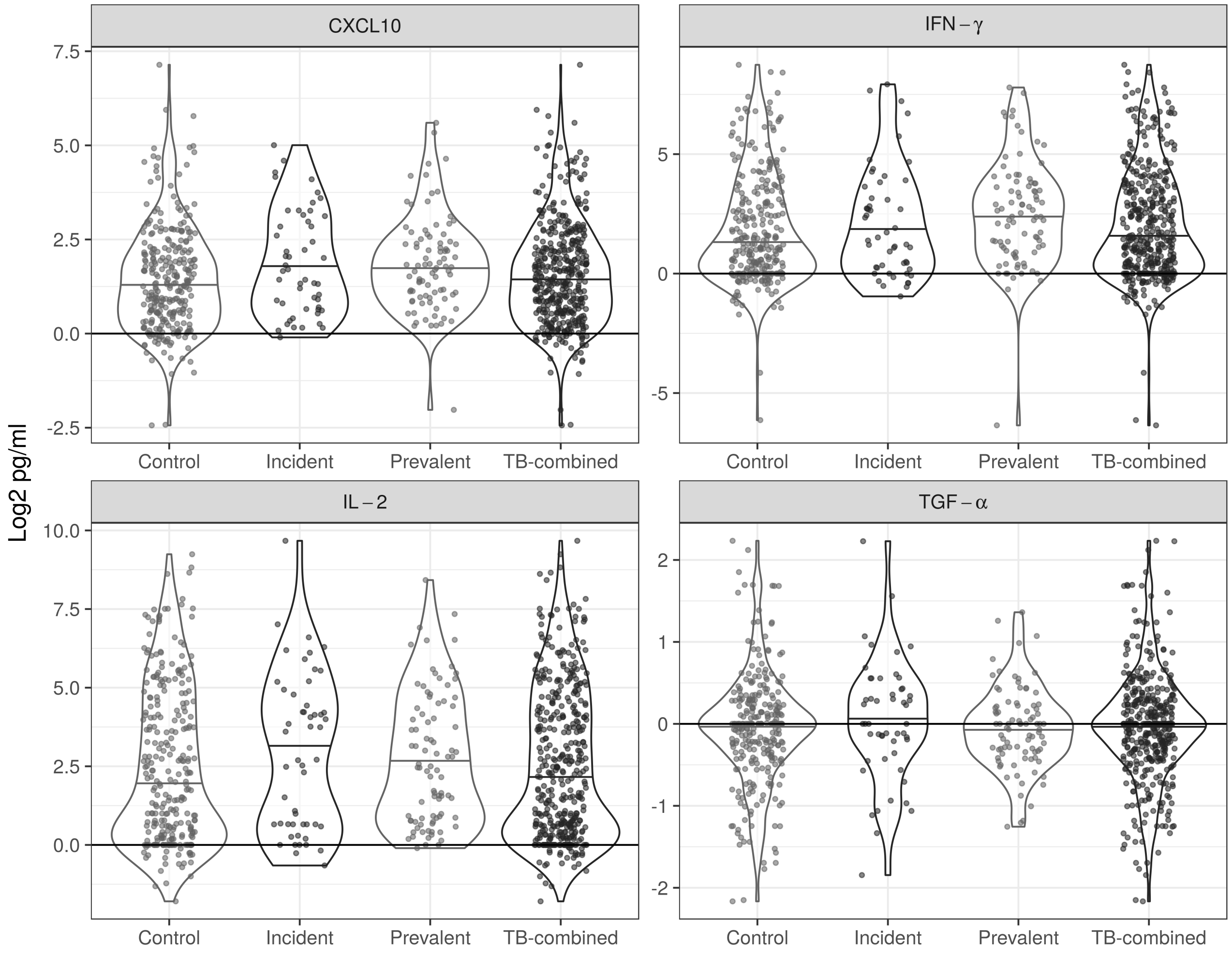

Figure S3

Weighted correlation networks in (left to right) controls, incident and prevalent TB groups respectively using background corrected analyte values. Solid lines are indicative of positive correlations, dashed lines indicate negative correlations and strength of correlation is indicated by thickness of line.

Control Ag-nil | Incident Ag-nil | Prevalent Ag-nil

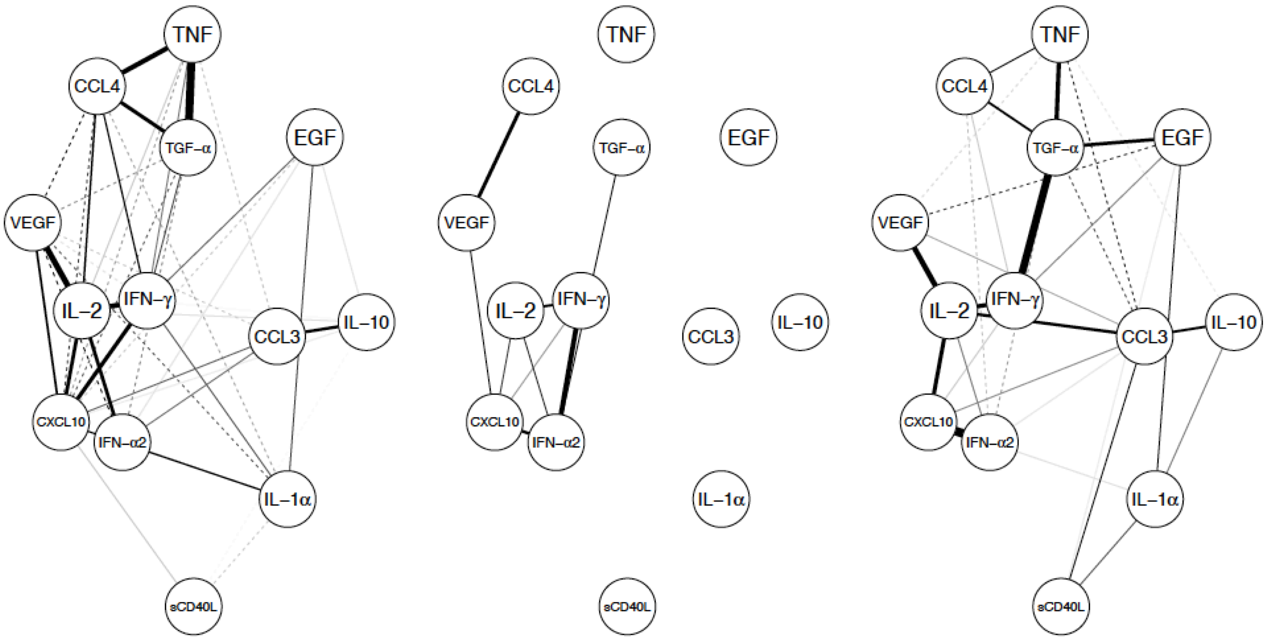

Figure S4

Receiver operator curves for sensitivity comparisons using the nil data and penalised regression models. Each curve represents an independent cross-validated predictive model with different tuning parameters.

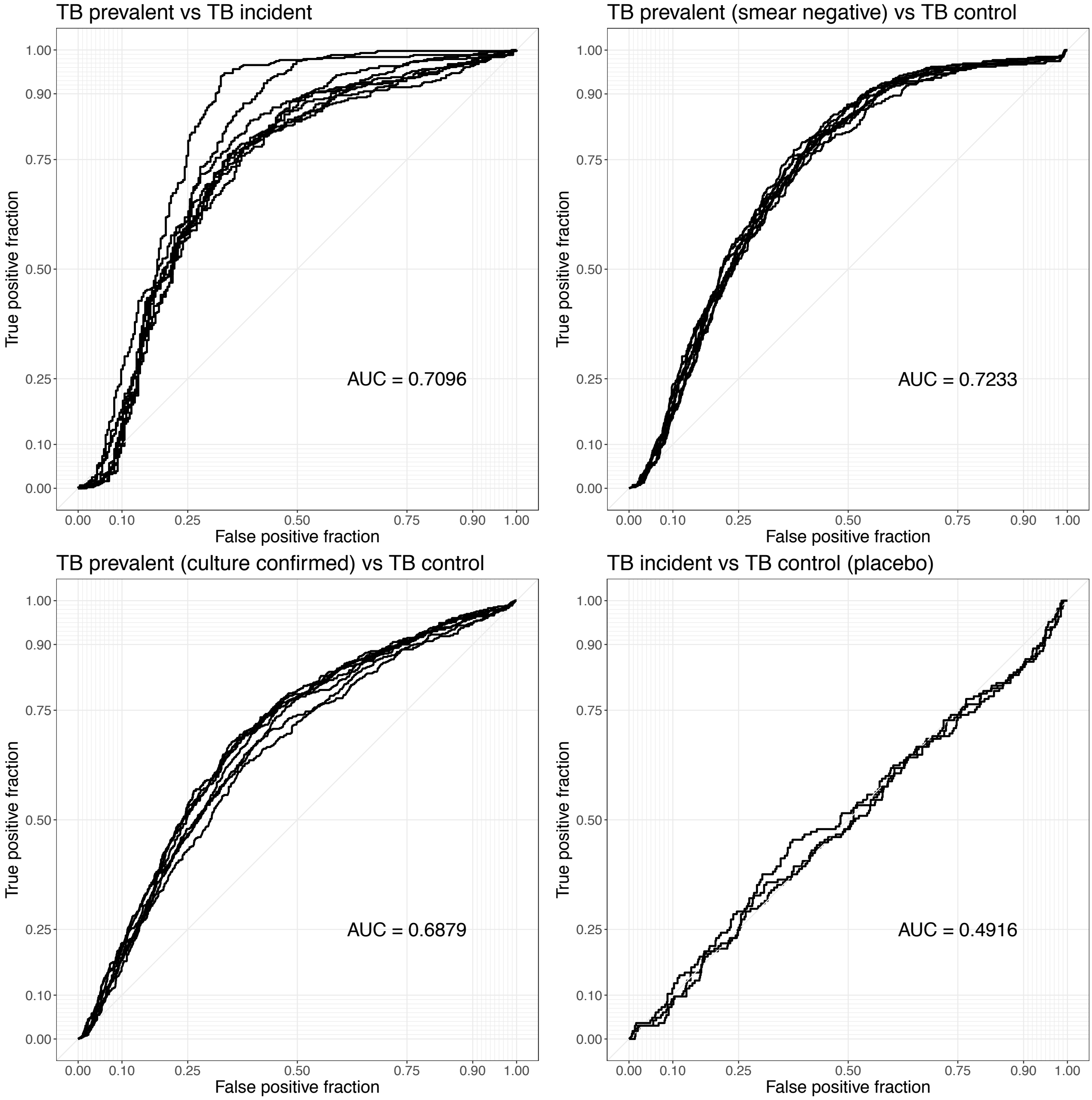

References for online supplement:

Sacha Epskamp, Angelique O. J. Cramer, Lourens J. Waldorp, Verena D. Schmittmann, Denny Borsboom (2012). qgraph: Network Visualizations of Relationships in Psychometric Data. Journal of Statistical Software, 48(4), 1-18. URL http://www.jstatsoft.org/v48/i04/.

Max Kuhn. Contributions from Jed Wing, Steve Weston, Andre Williams, Chris Keefer, Allan Engelhardt, Tony Cooper, Zachary Mayer, Brenton Kenkel, the R Core Team, Michael Benesty, Reynald Lescarbeau, Andrew Ziem, Luca Scrucca, Yuan Tang, Can Candan and Tyler Hunt. (2018). caret: Classification and Regression Training. R package version 6.0-80. https://CRAN.R-project.org/package=caret

Liaw A, Wiener M. Classification and Regression by randomForest. *R News* 2002; **2**(3), 18--22.
Friedman J, Hastie T, Tibshirani R. Regularization Paths for Generalized Linear Models via Coordinate Descent. *Journal of Statistical Software*, 2010; **33**(1):1–22

O'Garra A, Redford PS, McNab FW, Bloom CI, Wilkinson RJ, Berry MP. The immune response in tuberculosis. *Annu Rev Immunol*. 2013;31:475-527.

Beveridge NE, Price DA, Casazza JP, Pathan AA, Sander CR, Asher TE, Ambrozak DR, Precopio ML, Scheinberg P, Alder NC, Roederer M, Koup RA, Douek DC, Hill AV, McShane H. Immunisation with BCG and recombinant MVA85A induces long-lasting, polyfunctional *Mycobacterium tuberculosis*-specific CD4+ memory T lymphocyte populations. *Eur J Immunol*. 2007;37(11):3089-100.

Ruhwald M, Dominguez J, Latorre I, Losi M, Richeldi L, Pasticci MB, Mazzolla R, Goletti D, Butera O, Bruchfeld J, Gaines H, Gerogianni I, Tuuminen T, Ferrara G, Eugen-Olsen J, Ravn P; TBNET. A multicentre evaluation of the accuracy and performance of IP-10 for the diagnosis of infection with M. *tuberculosis.Tuberculosis*. 2011;91(3):260-7.

Ruhwald M, Bjerregaard-Andersen M, Rabna P, Kofoed K, Eugen-Olsen J, Ravn P. CXCL10/IP-10 release is induced by incubation of whole blood from tuberculosis patients with ESAT-6, CFP10 and TB7.7. *Microbes Infect*. 2007;9(7):806-12.

Chegou NN, Black GF, Kidd M, van Helden PD, Walzl G. Host markers in QuantiFERON supernatants differentiate active TB from latent TB infection: preliminary report. *BMC Pulm Med*. 2009 16;9:21.

Harari A, Rozot V, Bellutti Enders F, Perreau M, Stalder JM, Nicod LP, Cavassini M, Calandra T, Blanchet CL, Jaton K, Faouzi M, Day CL, Hanekom WA, Bart PA, Pantaleo G. Dominant TNF-α+ Mycobacterium tuberculosis-specific CD4+ T cell responses discriminate between latent infection and active disease. *Nat Med*. 2011;17(3):372-6.
